# Inverse associations between serum levels of secreted frizzled-related protein-5 (SFRP5) and multiple cardiometabolic risk factors: KORA F4 study

**DOI:** 10.1186/s12933-017-0591-x

**Published:** 2017-08-29

**Authors:** Maren Carstensen-Kirberg, Julia M. Kannenberg, Cornelia Huth, Christa Meisinger, Wolfgang Koenig, Margit Heier, Annette Peters, Wolfgang Rathmann, Michael Roden, Christian Herder, Barbara Thorand

**Affiliations:** 1 Institute for Clinical Diabetology, German Diabetes Center, Leibniz Center for Diabetes Research at Heinrich Heine University Düsseldorf, Auf’m Hennekamp 65, 40225 Düsseldorf, Germany; 2 German Center for Diabetes Research (DZD), München-Neuherberg, Germany; 3 Institute of Epidemiology II, Helmholtz Zentrum München, German Research Center for Environmental Health, Neuherberg, Germany; 4 Ludwig-Maximilians-Universität München, UNIKA-T Augsburg, Augsburg, Germany; 5 Deutsches Herzzentrum München, Technische Universität München, Munich, Germany; 6 German Center for Cardiovascular Research (DZHK), Partner Site Munich Heart Alliance, Munich, Germany; 7 Institute for Biometrics and Epidemiology, German Diabetes Center, Leibniz Center for Diabetes Research at Heinrich Heine University Düsseldorf, Düsseldorf, Germany; 8 Division of Endocrinology and Diabetology, Medical Faculty, Heinrich Heine University, Düsseldorf, Germany

**Keywords:** Inflammation, Secreted frizzled-related protein-5, Cytokine, Type 2 diabetes, Cardiometabolic risk

## Abstract

**Aims:**

Secreted frizzled-related protein (Sfrp)5 has beneficial effects on insulin sensitivity, inflammation and cardiovascular risk in different mouse models, but its relevance for cardiometabolic diseases in humans is controversial. We aimed to characterise associations of circulating SFRP5 with cardiometabolic risk factors and prediabetes/type 2 diabetes in a large population-based cohort.

**Methods:**

Cross-sectional associations between serum SFRP5 and cardiometabolic risk factors as well as prediabetes/type 2 diabetes were investigated in 1096 participants aged 62–81 years from the German KORA F4 study, of whom 666 had prediabetes or type 2 diabetes. Multivariable linear regression models were adjusted for potential confounders including age, sex, body mass index (BMI), lifestyle factors, lipids, hypertension, kidney function and myocardial infarction.

**Results:**

Higher serum SFRP5 levels were associated with lower HbA1c, BMI, systolic blood pressure, estimated glomerular filtration rate and high-sensitivity C-reactive protein levels and with higher levels of high-density lipoprotein cholesterol and adiponectin in the fully adjusted model (all *P* < 0.009). In contrast, favourable associations between SFRP5 and glycaemia, insulin, insulin resistance and other cardiometabolic risk factors were attenuated after adjustment for BMI. Serum SFRP5 levels were lower in participants with prediabetes or type 2 diabetes [(median (25th; 75th percentile) 48.8 (35.5; 65.7) ng/ml] compared to participants with normal glucose tolerance [55.9 (42.6; 69.6) ng/ml] (*P* < 0.001). In the fully adjusted model, higher SFRP5 was associated with lower odds of prediabetes/type 2 diabetes [OR (95% CI) (0.72 (0.58; 0.89)) per doubling of SFRP5, *P* < 0.01].

**Conclusions:**

Higher serum SFRP5 was inversely associated with multiple risk factors for type 2 diabetes and cardiovascular diseases. However, BMI represents a strong confounder of some of these associations. Higher circulating SFRP5 was also associated with lower odds of prediabetes/type 2 diabetes, and this association was independent of BMI. Thus, SFRP5 emerges as novel biomarker that merits further research in the context of prevention of cardiometabolic diseases.

**Electronic supplementary material:**

The online version of this article (doi:10.1186/s12933-017-0591-x) contains supplementary material, which is available to authorized users.

## Background

The secreted frizzled-related protein 5 (SFRP5) belongs to the SFRP family, which comprises five members in humans, SFRP1-5, and is the largest family of Wnt inhibitors [1]. However, SFRPs also antagonise one another´s activity, are able to bind to Frizzled receptors and interact with molecules outside of the Wnt signaling pathway [1]. SFRP5 is a secreted protein which is produced by several tissues such as visceral, subcutaneous and pericardial adipose tissue, liver, heart, mononuclear blood cells and pancreatic islets [2–8].

Obese mice lacking Sfrp5 have impaired glucose clearance and reduced insulin sensitivity. Moreover, these mice are characterised by an increased content of macrophages and pro-inflammatory proteins in adipose tissue [9]. In mice that underwent ischaemia reperfusion injury, Sfrp5 mRNA was reduced in pericardial adipose tissue. The knockout of Sfrp5 in mice led to an increased size of myocardial infarction, a higher myocyte apoptosis rate and enhanced mRNA levels of pro-inflammatory proteins in the ischaemic area [8].

In humans, data on SFRP5 and cardiometabolic diseases are controversial. Cross-sectional studies including people with and/or without type 2 diabetes found that circulating SFRP5 showed a positive [10], an inverse [11–13] or no association [14] with insulin resistance. In two hospital-based studies serum SFRP5 was inversely associated with the metabolic syndrome and the severity of coronary artery disease [13, 15]. However, data from population-based studies on SFRP5 and cardiometabolic risk are currently not available.

Therefore, this study aimed (a) to characterise the associations of serum SFRP5 levels with cardiometabolic risk factors, (b) to determine the relationship between SFRP5 and the odds of prediabetes/type 2 diabetes, and (c) to investigate to what extent these associations were independent of obesity and other potential confounders.

## Study participants and methods

### Study population

The study is based on data from the Cooperative Health Research in the Region of Augsburg (KORA) F4 study (2006–2008), a follow-up examination of the population-based KORA S4 study (1999–2001) conducted in Augsburg (Germany) and two surrounding counties. The design of the KORA studies has been described before [16, 17]. Briefly, study participants were invited from the city of Augsburg and two adjacent counties based on the survey sampling method of the former World Health Organisation Monitoring of Trends and Determinants of Cardiovascular Disease (WHO MONICA) project [17]. Within each selected community in the study area, age and sex-stratified samples were drawn with four of these strata including men and women aged 55–74 years. This cross-sectional analysis uses data from all study participants between 62 and 81 years of age in the KORA F4 study (*n* = 1161). Additional file 1: Figure S1 provides an overview of the study design, and Additional file 1: Table S1 compares participants and non-participants in KORA F4. In our analysis of the KORA F4 study sample, we excluded persons with unclear glucose tolerance status due to missing values for fasting and/or 2-h glucose (*n* = 33), type 1 diabetes (*n* = 2), drug-induced diabetes (*n* = 1) or other missing covariables (*n* = 29), resulting in a sample size of 1096 individuals.

### Anthropometric measurements

Height, body weight, waist circumference, systolic and diastolic blood pressure were measured as described [16, 17]. Body mass index (BMI) was calculated as body weight (kg)/[height (m)]^2^. Actual hypertension was defined as blood pressure of 140/90 mmHg or higher, or use of antihypertensive medication given that the study participants were aware of being hypertensive.

### Assessment of glucose tolerance and diabetes status

Known type 2 diabetes was defined as self-report of a previous diagnosis of type 2 diabetes that could be validated by the responsible physician, or as current use of glucose-lowering agents. All study participants without known type 2 diabetes were assigned to a standard 75-g oral glucose tolerance test (OGTT). Glucose tolerance categories were defined using fasting and 2-h glucose concentrations according to criteria of the American Diabetes Association [18]. Prediabetes was defined as impaired fasting glucose (IFG), impaired glucose tolerance (IGT) or combined IFG/IGT.

Glucose and insulin levels and HbA1c were measured as previously described [19]. Insulin sensitivity was assessed by calculation of the homeostasis model assessment of insulin resistance (HOMA-IR) based on fasting glucose and insulin levels and the whole-body insulin sensitivity index (ISI [composite]) based on both fasting and 2-h levels of glucose and insulin [19–21]. Fasting beta-cell function was estimated using HOMA-β [20].

### Assessment of further metabolic, lifestyle and immunological variables

Serum lipid levels were measured as described [19]. Kidney function was assessed from the estimated glomerular filtration rate (eGFR) using the chronic kidney disease epidemiology (CKD-EPI) creatinine equation [22, 23].

Trained medical interviewers collected information on medical history, physical activity, smoking, alcohol consumption and use of medication [16]. Study participants were classified as physically active if they reported >1 h of physical activity per week during leisure time in either summer or winter. Smoking status was defined as never, former or current smoking. Alcohol consumption was classified as none, moderate or high for an alcohol intake of 0, 0.1–39.9 or ≥40 g/day for men and 0, 0.1–19.9 or ≥20 g/day for women, respectively.

Plasma levels of high-sensitivity C-reactive protein (hsCRP) and serum levels of interleukin (IL)-6, IL-1 receptor antagonist (IL-1RA) and total adiponectin were measured as described [24].

### Quantification of SFRP5 serum levels

SFRP5 levels were measured in serum samples between 08/2016 and 09/2016 that had been continuously stored at −80 °C between blood sampling and analysis. Measurements were carried out using the Enzyme-linked Immunosorbent Assay Kit for Secreted Frizzled Related protein 5 (SFRP5) from Cloud-Clone (Houston, TX; previously USCN, Wuhan, China). The limit of detection (LOD) was 1.56 ng/ml. All sera yielded SFRP5 levels above the LOD. Coefficients of variation (CV) were assessed using three control sera measured in duplicates on 16 plates. Mean intra- and inter-assay CV were 6.4 and 18.6%, respectively.

### Statistical analysis

Characteristics of the study population are given stratified by quarters of serum SFRP5 and with age and sex-adjusted *P* values from linear regression analysis for associations between SFRP5 and these characteristics.

Cross-sectional associations between log_2_(SFRP5) and cardiometabolic risk factors were estimated by multivariable linear regression analysis using the following predefined set of potential confounders from previous KORA analyses [19, 25]:

- Model 1, adjusted for age and sex.
- Model 2, model 1 + smoking status (current/former/never), alcohol consumption, physical activity (active/inactive).
- Model 3, model 2 + BMI.
- Model 4, model 3 + hypertension (yes/no), HDL cholesterol, LDL cholesterol, triglycerides, lipid-lowering medication (yes/no), prevalent myocardial infarction (yes/no), eGFR.

Age, alcohol consumption, BMI, HDL cholesterol, LDL cholesterol, triglycerides and eGFR entered the models as continuous variables. In a sensitivity analysis, we replaced BMI with waist circumference in models 3 and 4.

Associations of log_2_ (SFRP5) with prediabetes/diabetes or separate glucose tolerance status groups were assessed by logistic and multinomial logistic regression analysis, respectively, with individuals with normal glucose tolerance as reference group adjusting for the aforementioned covariables.

Associations between HbA1c, time since diabetes diagnosis and glucose-lowering medication and log_2_ (SFRP5) were also estimated using linear regression analyses.

We checked the degree of multicollinearity in all models by calculating the variance inflation factors (VIF). Multicollinearity is considered high for VIF > 10, but VIF were <1.7 in all analyses.

All statistical analyses were performed with R version 3.3.3 (R Core Team, R Foundation for Statistical Computing, Vienna, Austria) and Python version 3.6.1 (Python Software Foundation, https://www.python.org/). A *P* value <0.05 was considered to indicate statistical significance.

## Results

Additional file 1: Table S1 gives a brief comparison of participants (*n* = 1161) and non-participants (*n* = 492) in the KORA F4 study. Non-participants were older and had higher systolic blood pressure and triglyceride levels, but did not differ in sex distribution, BMI, HbA1c, diastolic blood pressure and cholesterol levels.

Table 1 shows the characteristics of the study population after the exclusions described above (*n* = 1096) stratified by quarters of SFRP5 serum levels. Higher SFRP5 levels were associated with higher age, whereas no association with sex was observed. After initial adjustment for age and sex, higher SFRP5 levels were related to a more favourable cardiometabolic risk profile as evident by lower BMI and waist circumference, lower levels of fasting and 2-h glucose and insulin, lower HbA1c, higher insulin sensitivity and better glucose tolerance. Higher SFRP5 levels were also associated with lower systolic and diastolic blood pressure, higher HDL cholesterol, lower triglycerides, lower levels of biomarkers of subclinical inflammation (hsCRP, IL-6, IL-1RA) and higher adiponectin levels. No associations were found between SFRP5 and fasting beta-cell function, the prevalence of hypertension, LDL cholesterol, use of lipid-lowering drugs, smoking, physical activity and alcohol consumption. However, higher SFRP5 levels were associated with decreased eGFR.

**Table 1:** Description of the KORA F4 study population stratified by quarters of SFRP5 serum concentrations

Variable	Quarter 1	Quarter 2	Quarter 3	Quarter 4	*P*
*n*	274	274	274	274	
SFRP5 (ng/ml)	29.48 (23.88; 33.71)	44.54 (41.02; 48.23)	58.92 (55.58; 63.24)	79.78 (72.17; 89.54)	<0.001
Age (years)	69.7 ± 5.1	70.3 ± 5.4	69.7 ± 5.3	71.2 ± 5.7	0.001
Sex (% male)	45.3	56.6	53.3	50.7	0.281
BMI (kg/m^2^)	30.6 ± 4.6	29 ± 4.3	28.1 ± 4.2	27.3 ± 4.0	<0.001
Waist circumference (cm)	102.2 ± 12.4	99.4 ± 12.0	97.3 ± 12.2	94.6 ± 11.1	<0.001
Fasting glucose (mmol/l)^a^	5.6 ± 0.6	5.6 ± 0.9	5.4 ± 0.6	5.4 ± 0.6	<0.001
2-h glucose (mmol/l)^a^	7.5 ± 2.2	7.2 ± 2.4	6.8 ± 2.1	7.1 ± 2.4	0.002
HbA1c (%)^a^	5.68 ± 0.37	5.66 ± 0.51	5.60 ± 0.33	5.55 ± 0.35	<0.001
Fasting insulin (µU/ml)^a,b^	6.0 (4.2; 10.4)	5.0 (3.3; 8.8)	4.6 (3.2; 7.5)	4.6 (3.0; 7.4)	0.007
2-h insulin (µU/ml)^a,b^	65.7 (38.3; 105.9)	57.2 (33.7; 91.2)	50.0 (28.2; 77.7)	49.1 (25.1; 82.5)	0.001
HOMA-IR^a,b^	1.46 (1.00; 2.68)	1.22 (0.79; 2.23)	1.09 (0.73; 1.95)	1.07 (0.69; 1.87)	0.002
ISI (composite) (1/((mmol/l) × (pmol/l)))^a,b^	12.74 (8.01; 22.52)	16.77 (9.22; 25.63)	18.74 (11.18; 29.59)	18.64 (10.41; 35.77)	<0.001
HOMA-β^a^	59.13 (41.51; 108.17)	52.46 (33.88; 87.64)	52.97 (35.95; 87.21)	50.93 (33.71; 90.09)	0.170
Glucose tolerance status: NGT/IFG/IGT/IFG and IGT/ndT2D/T2D (%)	29.2/19.7/12.8/12.4/6.2/19.7	34.7/23.7/9.9/10.9/5.8/15.0	48.9/15.7/7.3/11.3/5.1/11.7	44.2/16.1/9.9/10.2/9.1/10.6	<0.001
Systolic blood pressure (mmHg)^c^	131.3 ± 17.6	130.2 ± 15.9	128 ± 19.5	125.4 ± 20.8	<0.001
Diastolic blood pressure (mmHg)^c^	76.8 ± 9.4	76.8 ± 8.8	75.7 ± 9.8	74.0 ± 9.4	0.009
Hypertension (%)	61.7	64.2	60.6	63.9	0.782
LDL cholesterol (mmol/l)^d^	3.81 ± 0.86	3.78 ± 0.87	3.84 ± 0.96	3.75 ± 0.89	0.334
HDL cholesterol (mmol/l)^d^	1.4 ± 0.38	1.42 ± 0.35	1.45 ± 0.35	1.53 ± 0.40	<0.001
Triglycerides (mmol/l)^b,d^	1.36 (0.92; 1.89)	1.25 (0.98; 1.80)	1.27 (0.95; 1.72)	1.23 (0.89; 1.64)	0.026
Use of lipid-lowering drugs (%)	22.6	25.5	24.1	25.7	0.373
eGFR (ml/min per 1.73 m^2^)	78.9 ± 13.0	77.5 ± 14.9	76.8 ± 14.9	72.4 ± 16.4	<0.001
Smoking (never/former/current) (%)	49.3/43.4/7.3	49.3/42.0/8.8	46.0/46.0/8.0	50.7/43.1/6.2	0.992
Physically active (%)	46.0	51.1	52.2	50.7	0.270
Alcohol consumption (none/moderate/high) (%)	36.5/53.6/9.9	27.4/62.4/10.2	33.9/56.9/9.1	29.9/61.3/8.8	0.170
hs C-reactive protein (mg/l)^b^	2.25 (1.04; 4.38)	1.46 (0.89; 2.91)	1.38 (0.75; 2.82)	1.39 (0.62; 2.75)	<0.001
IL-6 (pg/ml)^b^	1.81 (1.21; 2.67)	1.63 (1.17; 2.45)	1.57 (1.08; 2.34)	1.52 (0.98; 2.26)	0.001
IL-1 receptor antagonist (pg/ml)^b^	344 (263; 446)	300 (235; 391)	303 (229; 379)	296 (232; 392)	<0.001
Adiponectin (µg/ml)^b^	9.43 (6.13; 14.02)	8.68 (6.07; 13.59)	10.44 (7.37; 14.86)	11.80 (8.27; 18.08)	<0.001

Data are given as mean ± SD, median and 25th; 75th percentiles or percentages, unless indicated otherwise. *P* values are adjusted for age and sex using linear regression analysis. The analysis for age is adjusted for sex only, the analysis for sex is adjusted for age only

*eGFR* estimated glomerular filtration rate, *HDL* high-density lipoprotein, *HOMA* homeostasis model assessment, *hs* high-sensitivity, *IFG* impaired fasting glucose, *IGT* impaired glucose tolerance, *IL* interleukin, *ISI* insulin sensitivity index, *LDL* low-density lipoprotein, *ndT2D* newly diagnosed type 2 diabetes, *NGT* normal glucose tolerance, *T2D* type 2 diabetes

^a^Individuals with known type 2 diabetes (*n* = 156) excluded

^b^Variables were log_2_-transformed for the linear regression analysis

^c^Individuals with anti-hypertensive medication (*n* = 627) excluded

^d^Individuals with lipid-lowering medication (*n* = 270) excluded

Multivariable linear regression analyses showed that the aformentioned associations between SFRP5 levels and cardiometabolic risk factors (Tables 1 and 2, model 1) were largely unaffected by adjustment for lifestyle factors (physical activity, smoking, alcohol consumption; model 2). Further adjustment for BMI (model 3) attenuated or even abolished most of these associations, whereas additional adjustment for metabolic factors (HDL cholesterol, LDL cholesterol, triglycerides, lipid-lowering medication, hypertension), history of myocardial infarction and kidney function (model 4) had almost no impact on the strength of the associations. In the fully adjusted model, higher SFRP5 levels remained associated with lower HbA1c, lower BMI, lower systolic blood pressure, higher HDL cholesterol, lower eGFR, lower hsCRP and higher adiponectin levels (all *P* ≤ 0.009).

**Table 2:** Associations between SFRP5 and cardiometabolic risk factors in the KORA F4 study

Variable	Model 1		Model 2		Model 3		Model 4	
	β (95% CI)	*P*	β (95% CI)	*P*	β (95% CI)	*P*	β (95% CI)	*P*
Fasting glucose (mmol/l)^a^	*−0.12* (*−0.18*; *−0.06*)	*<0.001*	*−0.12* (*−0.18*; *−0.05*)	*<0.001*	−0.05 (−0.11; 0.02)	0.162	−0.06 (−0.12; 0.00)	0.061
2-h glucose (mmol/l)^a^	−*0.33* (−*0.53*; *−0.12*)	*0.002*	*−0.30* (*−0.51*; *−0.10*)	*0.004*	−0.11 (−0.32; 0.10)	0.301	−0.13 (−0.34; 0.08)	0.228
HbA1c (%)^a^	−*0.08* (*−0.12*; *−0.05*)	*<0.001*	*−0.08* (*−0.12*; *−0.05*)	*<0.001*	*−0.06* (*−0.10*; *−0.03*)	*0.001*	*−0.07* (*−0.11*; *−0.03*)	*<0.001*
Fasting insulin (µU/ml)^a,b^	−*0.17* (−*0.29*; −*0.05*)	*0.007*	*−0.16* (*−0.28*; *−0.04*)	*0.010*	0.05 (−0.07; 0.16)	0.446	0.06 (−0.06; 0.18)	0.367
2-h insulin (µU/ml)^a,b^	−*0.21* (−*0.33*; −*0.09*)	*0.001*	*−0.20* (*−0.31*; *−0.08*)	*0.001*	−0.03 (−0.14; 0.09)	0.647	−0.03 (−0.15; 0.08)	0.571
HOMA-IR^a,b^	−*0.20* (−*0.33*; −*0.07*)	*0.002*	*−0.19* (*−0.32*; *−0.06*)	*0.003*	0.04 (−0.09; 0.16)	0.573	0.04 (−0.08; 0.16)	0.518
ISI (composite) (1/((mmol/l) × (pmol/l)))^a,b^	*0.25* (*0.13*; *0.37*)	*<0.001*	*0.24* (*0.12*; *0.36*)	*<0.001*	0.02 (−0.09; 0.14)	0.707	0.03 (−0.09; 0.14)	0.667
HOMA-β^a^	−0.08 (−0.20; 0.03)	0.170	−0.08 (−0.19; 0.04)	0.206	0.08 (−0.04; 0.19)	0.207	0.09 (−0.03; 0.21)	0.126
BMI (kg/m^2^)	*−1.80* (*−2.16*; *−1.44*)	*<0.001*	*−1.77* (*−2.12*; *−1.42*)	*<0.001*	N/A	N/A	*−1.56* (*−1.90; −1.23*)	*<0.001*
Waist circumference (cm)	*−4.30* (*−5.21*; *−3.40*)	*<0.001*	*−4.23* (*−5.12*; *−3.34*)	*<0.001*	−0.27 (−0.71; 0.17)	0.229	−0.17 (−0.62; 0.28)	0.451
Systolic blood pressure (mmHg)^c^	*−4.19* (*−6.46*; *−1.92*)	*<0.001*	*−3.99* (*−6.28*; *−1.70*)	*0.001*	*−3.36 (−5.76; −0.95*)	*0.007*	*−3.40 (−5.83; −0.97*)	*0.006*
Diastolic blood pressure (mmHg)^c^	*−1.56* (*−2.73*; *−0.40*)	*0.009*	*−1.49* (*−2.67*; *−0.32*)	*0.013*	−0.95 (−2.18; 0.28)	0.131	−1.07 (−2.31; 0.16)	0.089
HDL cholesterol (mmol/l)^d^	*0.09 (0.05; 0.12)*	*<0.001*	*0.08* (*0.05*; *0.12*)	*<0.001*	0.03 (0.00; 0.06)	0.070	*0.04* (*0.01*; *0.08*)	*0.009*
LDL cholesterol (mmol/l)^d^	−0.04 (−0.13; 0.04)	0.334	−0.04 (−0.13; 0.04)	0.346	−0.06 (−0.15; 0.04)	0.229	−0.05 (−0.14; 0.04)	0.302
Triglycerides (mmol/l)^b,d^	*−0.08* (*−0.15*; *−0.01*)	*0.026*	*−0.08 (−0.15; −0.01)*	*0.029*	0.01 (−0.06; 0.08)	0.690	−0.02 (−0.09; 0.05)	0.646
Estimated glomerular filtration rate (ml/min per 1.73 m^2^)^e^	*−2.67* (*−3.80*; *−1.54*)	*<0.001*	*−2.70 (−3.82; −1.58)*	*0.001*	*−3.74* (*−4.89*; *−2.59*)	*<0.001*	*−3.77* (*−4.91*; *−2.63*)	*<0.001*
hsCRP (mg/l)^b^	*−0.32* (*−0.45*; *−0.20*)	*<0.001*	*−0.31 (−0.44; −0.19)*	*<0.001*	*−0.16* (*−0.28*; *−0.04*)	*0.012*	*−0.18* (*−0.31*; *−0.06*)	*0.005*
IL-6 (pg/ml)^b^	*−0.13* (*−0.21*; *−0.05*)	*0.001*	*−0.13 (−0.20; −0.05)*	*0.001*	−0.04 (−0.12; 0.04)	0.328	−0.05 (−0.13; 0.03)	0.328
IL-1RA (pg/ml)^b^	*−0.11* (*−0.16*; *−0.06*)	*<0.001*	*−0.11 (−0.16; −0.06)*	*<0.001*	−0.01 (−0.06; 0.04)	0.700	−0.02 (−0.07; 0.03)	0.484
Adiponectin (µg/ml)^b^	*0.19* (*0.13*; *0.25*)	*<0.001*	*0.19* (*0.13*; *0.25*)	*<0.001*	*0.13* (*0.06*; *0.19*)	*<0.001*	*0.09* (*0.03; 0.15*)	*0.003*

Regression coefficients *β* and 95% CI are standardised to a doubling in SFRP5 levels in linear regression analysis. Italics print indicates significant associations (*P* < 0.05)

*Model 1* adjusted for age, sex, *Model 2* model 1 + physical activity, smoking, alcohol consumption, *Model 3* model 2 + BMI, *Model 4* model 3 + HDL cholesterol, LDL cholesterol, triglycerides, lipid-lowering medication, hypertension, history of myocardial infarction, estimated glomerular filtration rate

^a^Individuals with known type 2 diabetes (*n* = 156) excluded

^b^Variables were log_2_-transformed for the linear regression analysis

^c^Individuals with anti-hypertensive medication (*n* = 627) excluded, no adjustment for hypertension in model 4

^d^Individuals with lipid-lowering medication (*n* = 270) excluded, no adjustment for HDL cholesterol, LDL cholesterol, triglycerides and lipid-lowering medication in model 4

^e^No adjustment for eGFR in model 4

A sensitivity analysis adjusting for waist circumference instead of BMI yielded similar results for models 3 and 4, but the inverse associations of SFRP5 with fasting glucose and with diastolic blood pressure reached significance (*P* = 0.033 and *P* = 0.046, respectively) in the fully adjusted model (data not shown).

Individuals with prediabetes or type 2 diabetes (total *n* = 666) had lower SFRP5 serum levels than individuals with normal glucose tolerance (*n* = 430, *P* < 0.001; Table 3). Logistic regression analysis showed that the odds of having prediabetes or diabetes was decreased by 40% per doubling of SFRP5 (OR [95% CI] 0.60 [0.50; 0.73], *P* < 0.001 in both models 1 and 2 adjusting for age, sex and lifestyle factors). This inverse association was slightly attenuated by further adjustment for BMI (model 3; *P* = 0.003), but remained significant in the fully adjusted model 4 (OR [95% CI] 0.72 [0.58; 0.89], *P* = 0.002; Table 3). When the group of individuals with prediabetes/diabetes was subdivided into smaller groups of individuals with IFG, IGT, combined IFG/IGT, newly diagnosed type 2 diabetes and known type 2 diabetes, SFRP5 was inversely associated with the odds of IFG, IGT and known type 2 diabetes after full adjustment (Additional file 1: Table S2). In the subgroup with type 2 diabetes, neither glycaemic control (assessed by HbA1c), duration since the diagnosis of type 2 diabetes nor the type of glucose-lowering medication showed any association with SFRP5 levels (all *P* > 0.05, Additional file 1: Table S3).

**Table 3:** Association between SFRP5 serum concentrations and prediabetes/type 2 diabetes

Group	*n*	SFRP5 levels (ng/ml)	Model 1	Model 2	Model 3	Model 4
			OR (95% CI)	OR (95% CI)	OR (95% CI)	OR (95% CI)
Normal glucose tolerance	430	55.9 (42.6, 69.6)	1	1	1	1
Prediabetes/type 2 diabetes	666	48.8 (35.5, 65.7)***	0.60 (0.50; 0.73)***	0.60 (0.50; 0.73)***	0.75 (0.61; 0.91)**	0.72 (0.58; 0.89)**

The prediabetes/type 2 diabetes group comprised all individuals with impaired fasting glucose and/or impaired glucose tolerance or with newly diagnosed or known type 2 diabetes. SFRP5 levels are given as median (25th; 75th percentiles) and were log_2_-transformed for the logistic regression analysis, so that OR (95% CI) refer to a doubling in SFRP5 levels

*Model 1* adjusted for age, sex, *Model 2* model 1 + physical activity, smoking, alcohol consumption, *Model 3* model 2 + BMI, *Model 4* model 3 + HDL cholesterol, LDL cholesterol, triglycerides, lipid-lowering medication, hypertension, history of myocardial infarction, estimated glomerular filtration rate

** *P* < 0.01, *** *P* < 0.001

## Discussion

This study has two main findings: (1) circulating SFRP5 showed inverse associations with multiple risk factors for type 2 diabetes and cardiovascular diseases and (2) higher serum SFRP5 was associated with a lower odd of prediabetes/type 2 diabetes.

### SFRP5 and type 2 diabetes

This is the first study that investigated the association between SFRP5, prediabetes and type 2 diabetes in a large population-based study and controlled for a range of potentially confounding factors. We extend the current literature by using both fasting and dynamic measures of insulin sensitivity. Our data shed light on the currently controversial data on SFRP5 and type 2 diabetes by demonstrating that higher serum SFRP5 was significantly linked to lower odds of prediabetes/type 2 diabetes (as combined group of individuals with impaired glucose regulation) even after adjustment for age, sex, BMI, lifestyle factors and risk factors for cardiovascular diseases.

Our study is in line with previous reports suggesting a protective role of Sfrp5 in the development of type 2 diabetes in mice [9] and with human studies demonstrating an inverse association between circulating SFRP5 and HOMA-IR, a fasting measure of insulin resistance [11–13, 15]. Additionally, two studies also reported lower Sfrp5 levels in patients with type 2 diabetes compared to individuals with normal glucose tolerance [11, 12]. However, it is in contrast to two human studies which found a positive [10] and no relationship [14] to HOMA-IR or higher Sfrp5 levels in patients with type 2 diabetes compared to prediabetic individuals, although no difference to the group with normal glucose tolerance was observed [14]. Different laboratory methods cannot explain the observed differences because this study and the aforementioned studies [10–15] used the same method to measure circulating SFRP5 (purchased from Cloud-Clone, previously distributed by USCN). However, the previous studies were based on small sample sizes, they were not population-based and differed in their adjustment for potential confounders. In particular the study with the opposing result of higher Sfrp5 levels in patients with type 2 diabetes was based on highly selected individuals with optimal glycaemic control (median HbA1c 5.8%) who cannot be considered representative for people with type 2 diabetes in the general population [14].

In contrast to the majority of epidemiological studies, the treatment of primary human adipocytes with recombinant SFRP5 reduced the phosphorylation levels of members of the insulin signaling pathway, which would argue for a detrimental role of SFRP5 for insulin sensitivity in these cells [26]. It may be possible that systemic and local effects of SFRP5 on insulin-sensitive cells are different, because the in vitro study concentrated on the impact of SFRP5 on adipocytes and did not consider the interaction with other cells from the adipose tissue as well as potential locally occurring binding partners.

### SFRP5 and other cardiovascular risk factors

The occurrence of cardiovascular diseases can be an important consequence of type 2 diabetes. In this study, we also investigated the association of serum SFRP5 with multiple cardiovascular risk factors in addition to glycaemia and prediabetes/type 2 diabetes. We found that higher serum SFRP5 was associated with lower systolic and diastolic blood pressure, higher HDL cholesterol and lower triglycerides. All associations were independent of age, sex and lifestyle factors. BMI attenuated all investigated relationships apart from those with systolic blood pressure and HDL cholesterol.

Previous findings were based on small sample sizes from hospital-based studies and included only people with Asian ethnicity with known differences in body fat distribution and adipokine levels compared to other ethnic groups. A recent hospital-based study found that circulating SFRP5 was associated with a lower odds of the metabolic syndrome irrespective of age, sex and risk factors for cardiometabolic diseases [15]. In this study population [15], circulating SFRP5 correlated inversely with systolic and diastolic blood pressure, triglycerides, free fatty acids and atherosclerotic index and positively with HDL cholesterol in a model adjusted for age and weight-to-hip ratio. In obese children with hypertension plasma SFRP5 was reduced, while lifestyle intervention did not only improve blood pressure and BMI but also increased plasma SFRP5 [27]. Another study found that serum SFRP5 was lower in patients with coronary artery disease compared to controls and correlated inversely with the severity of the disease [13]. In mice, Sfrp5 reduced myocardial infarction size and myocyte apoptosis in the heart after ischaemia/reperfusion injury. This might be partially mediated by inhibition of pro-inflammatory effects of the Wnt5a/JNK signaling in macrophages [8] which also plays an important role in adipose tissue inflammation [28] and endothelial dysfunction [29] and thus underlines the protective role of Sfrp5 on cardiovascular diseases. Collectively, these data demonstrate robust inverse associations between circulating SFRP5 and a range of cardiovascular risk factors in human cohorts using different study designs and populations, which are corroborated by data from animal models. However, data on cardiovascular outcomes are still very limited.

Interestingly, higher serum SFRP5 was associated with impaired renal function measured as estimated glomerular filtration rate. The link between SFRP5 and renal function has not been investigated in detail yet. One study reported that SFRP5 inhibited high phosphate-induced calcification of vascular smooth muscle cells which is a major cardiovascular risk factor in chronic kidney disease (CKD) [30]. However, another study showed that the *Sfrp5* gene is hypermethylated and downregulated in a mouse model of CKD, whereas injection of recombinant Sfrp5 inhibited Wnt signaling and attenuated renal fibrosis [31]. Currently, data on the association between SFRP5 and eGFR or albuminuria as measures of kidney function from other study populations are not available, so that further studies are required to corroborate or refute our finding.

### SFRP5 and subclinical inflammation

One important risk factor for type 2 diabetes and its comorbidities is subclinical inflammation [32]. In the current study, we found that higher circulating SFRP5 was associated with lower levels of the pro-inflammatory markers hsCRP and IL-6, and the anti-inflammatory protein IL-1Ra and with higher levels of adiponectin. BMI explained the associations between SFRP5, IL-6 and IL-1Ra, but not those with hsCRP and adiponectin. It might be surprising that high SFRP5 was associated with lower levels of the anti-inflammatory IL-1Ra which argues against an anti-inflammatory action of SFRP5 at a first glance. However, prospective studies clearly demonstrated that higher IL-1Ra levels were associated with a higher risk of type 2 diabetes and cardiovascular events [33–35]. This association may at least partially reflect a response to proatherogenic factors such as subclinical inflammation (including IL-1β-mediated processes, which upregulate IL-1Ra) and oxidative stress.

Previous human cross-sectional studies supported the anti-inflammatory phenotype of SFRP5. They found that circulating SFRP5 was associated inversely with hsCRP, leptin, IL-6, TNFα and IL-1β and positively with adiponectin [12, 13, 15, 36, 37]. On the cellular level, the treatment with recombinant SFRP5 reduced the release of IL-6 by 49% in vitro in TNFα-induced primary human adipocytes. In that study, SFRP5 did not influence the activation of JNK, but decreased the activation of NFκB [26]. In obese mice Sfrp5 reduced the levels of the pro-inflammatory proteins TNFα, IL-6 and MCP-1 and the content of macrophages in adipose tissue [9]. Collectively, these data indicate that SFRP5 may have anti-inflammatory effects which could mediate the relationship between higher SFRP5 and lower cardiometabolic risk.

### Impact of BMI on associations between SFRP5 and cardiometabolic risk factors

The most important confounder which influenced the associations between SFRP5 and risk factors for cardiometabolic diseases was BMI. In our study, high serum SFRP5 was associated with a lower BMI. This is in line with other human studies showing that circulating SFRP5 associated inversely with BMI [7, 13, 15, 36] and plasma levels of SFRP5 were decreased in obese people [11, 12]. Data from mouse studies reported that Sfrp5 levels depended on the duration of obesity. Mice fed a high-fat diet for a short time had increased levels of Sfrp5 in the adipose tissue, whereas a high-fat diet for a longer period of time decreased levels of Sfrp5 in the adipose tissue [9]. In a human study, caloric restriction led to a fast increase of serum levels of SFRP5 [5]. So far, it is not known whether SFRP5 plays a physiological role as mediator between BMI and obesity-associated comorbidities or between weight loss and an improved cardiometabolic risk profile.

### Strengths and limitations

The large sample size and the population-based design allowed a valid analysis of the association between circulating SFRP5 and cardiometabolic risk factors. An important limitation of our study is the cross-sectional design which precludes conclusions regarding the predictive value of circulating SFRP5 for the incidence of type 2 diabetes and cardiovascular events. Lower SFRP5 levels may be cause or consequence of prediabetes/type 2 diabetes. Our subanalyses in patients with type 2 diabetes showed no associations between metabolic control or medication and SFRP5 levels, which would argue against hyperglycaemia as cause of lower SFRP5. However, one study reported an increase of SFRP5 levels following glucose-lowering therapy [11], so that further studies are required to resolve the temporal relationship between changes in SFRP5 and changes in glucose metabolism. Additionally, participants and non-participants in the KORA F4 study showed some differences in their baseline characteristics in the population-based KORA S4 study, but these differences were small, so that we have no indication for a relevant selection bias due to loss to follow-up. Moreover, the study is based on older individuals of German descent which limits the generalisability of our observations to younger populations and people with different ethnic background.

## Conclusion

In the population-based KORA study, circulating SFRP5 was independently associated with BMI, HbA1c, systolic blood pressure, HDL cholesterol, eGFR, hsCRP and adiponectin which represent risk factors for type 2 diabetes and diabetes-related cardiovascular diseases. BMI partially explained the associations between serum SFRP5 and cardiometabolic risk factors. However, the association between high SFRP5 levels with lower odds of prediabetes/type 2 diabetes remained significant after adjustment for confounders including BMI. Thus, SFRP5 emerges as novel biomarker that merits further research in the context of prevention of cardiometabolic diseases.

## Additional file

**Additional file 1: Table S1.** Baseline characteristics (KORA S4, 1999-2001) of participants and non-participants in KORA F4 (2006-2008). **Table S2.** Association between SFRP5 serum concentrations and glucose tolerance status. **Table S3.** Association between SFRP5 serum concentrations and HbA1c, duration of diabetes and glucose-lowering medication in study participants with type 2 diabetes. **Figure S1.** Description of the study design.
