# Supplementary material for: Inverse associations between serum levels of secreted frizzled-related protein-5 (SFRP5) and multiple cardiometabolic risk factors: KORA F4 study
Source: Cardiovasc Diabetol. 2017 Aug 29;16:109. doi: 10.1186/s12933-017-0591-x (PMC5574239; doi:10.1186/s12933-017-0591-x)

**Table S1 Baseline characteristics (KORA S4, 1999-2001) of participants and non-participants in KORA F4 (2006-2008)**

| **Variable** | **Participants in KORA F4** | **Non-participants in KORA F4** | ***P*** |
| --- | --- | --- | --- |
| *n* | 1161 | 492 |  |
| Age (years) | 63.3 ± 5.4 | 65.8 ± 5.3 | <0.001 |
| Sex (% male) | 51.2 | 50.4 | 0.671 |
| BMI (kg/m²) | 28.4 ± 4.2 | 29.0 ± 4.8 | 0.074 |
| HbA1c (%) ^a^ | 5.7 ± 0.7 | 5.8 ± 0.8 | 0.278 |
| Systolic blood pressure (mmHg) | 135.2 ± 19.8 | 140.2 ± 22.0 | 0.002 |
| Diastolic blood pressure (mmHg) | 80.6 ± 10.5 | 80.6 ± 11.1 | 0.526 |
| LDL cholesterol (mmol/l) | 3.96 ± 1.01 | 3.93 ± 1.07 | 0.486 |
| HDL cholesterol (mmol/l) | 1.50 ± 0.42 | 1.46 ± 0.42 | 0.135 |
| Triglycerides (mmol/l) | 1.36 (0.98; 1.90) | 1.43 (1.04; 2.12) | 0.016 |

Data are given as mean ± SD, median and 25^th^; 75^th^ percentiles or percentages. *P* values are adjusted for age and sex using linear regression analysis. The analysis for age is adjusted for sex only, the analysis for sex is adjusted for age only.

BMI, body mass index; HDL, high-density lipoprotein; LDL, low-density lipoprotein.

**Table S2**  **Association between SFRP5 serum concentrations and glucose tolerance status**

| **Group** | ***n*** | **SFRP5 levels (ng/ml)** | **Model 1** | **Model 2** | **Model 3** | **Model 4** |
| --- | --- | --- | --- | --- | --- | --- |
|  |  |  | **OR (95% CI)** | **OR (95% CI)** | **OR (95% CI)** | **OR (95% CI)** |
| Normal glucose tolerance | 430 | 55.9 (42.6, 69.6) | 1 | 1 | 1 | 1 |
| Impaired fasting glucose (IFG) | 206 | 48.3 (36.9, 64.2) | 0.61 (0.47; 0.78) | 0.60 (0.47; 0.78) | 0.71 (0.55; 0.92) | 0.70 (0.53; 0.91) |
| Impaired glucose tolerance (IGT) | 109 | 48.3 (34.2, 66.9) | 0.59 (0.44; 0.81) | 0.59 (0.43; 0.80) | 0.63 (0.45; 0.86) | 0.63 (0.45; 0.88) |
| Combined IFG/IGT | 123 | 51.2 (36.0, 65.4) | 0.62 (0.46; 0.83) | 0.61 (0.46; 0.83) | 0.77 (0.57; 1.05) | 0.75 (0.54; 1.04) |
| Newly diagnosed type 2 diabetes | 72 | 55.6 (38.2, 70.0) | 0.74 (0.51; 1.07) | 0.74 (0.51; 1.07) | 0.98 (0.67; 1.43) | 0.98 (0.65; 1.48) |
| Known type 2 diabetes | 156 | 45.7 (33.8, 63.0) | 0.53 (0.41; 0.70) | 0.54 (0.41; 0.72) | 0.77 (0.57; 1.03) | 0.71 (0.52; 0.98) |

SFRP5 levels are given as median (25^th^; 75^th^ percentiles) and were log_2_-transformed for the multinomial logistic regression analysis, so that OR (95% CI) refer to a doubling in SFRP5 levels.

Model 1: adjusted for age, sex.

Model 2: model 1 + physical activity, smoking, alcohol consumption.

Model 3: model 2 + BMI.

Model 4: model 3 + HDL cholesterol, LDL cholesterol, triglycerides, lipid-lowering medication, hypertension, history of myocardial infarction, estimated glomerular filtration rate.

**Table S3**  **Association between SFRP5 serum concentrations and HbA1c, duration of diabetes and glucose-lowering medication in study participants with type 2 diabetes**

|  | **Newly diagnosed T2D (*n*=72)** |  | **Known T2D (n=156)** |  | **All T2D (n=228)** |  |
| --- | --- | --- | --- | --- | --- | --- |
|  | ***β* (95% CI)** | ***P*** | ***β* (95% CI)** | ***P*** | ***β* (95% CI)** | ***P*** |
| HbA1c (%) | -0.12 (-0.34; 0.10) | 0.261 | -0.06 (-0.17; 0.05) | 0.291 | -0.08 (-0.17; 0.01) | 0.091 |
| Time since diagnosis of diabetes (years)* | n/a | n/a | 0.01 (0.00; 0.02) | 0.174 | 0.00 (-0.01; 0.01) | 0.683 |
| Use of oral-antidiabetic drugs** | n/a | n/a | -0.07 (-0.31; 0.18) | 0.861 | n/a | n/a |
| Insulin use** | n/a | n/a | 0.00 (-0.27; 0.28) | 0.990 | n/a | n/a |

Regression coefficients *β* and 95% CI are standardised to a doubling in SFRP5 levels in linear regression analysis. All results are adjusted for age, sex and BMI. n/a, not applicable; T2D, type 2 diabetes.

*Set as 0 years for newly diagnosed T2D when analysing all study participants with T2D.

**Analysis restricted to individuals with known T2D. Use of oral anti-diabetic drugs in *n*=103, insulin use in *n*=35, use of at least one of the aforementioned medications in *n*=116. Both types of medication were assessed in the same model.

**Figure S1** **Description of the study design**


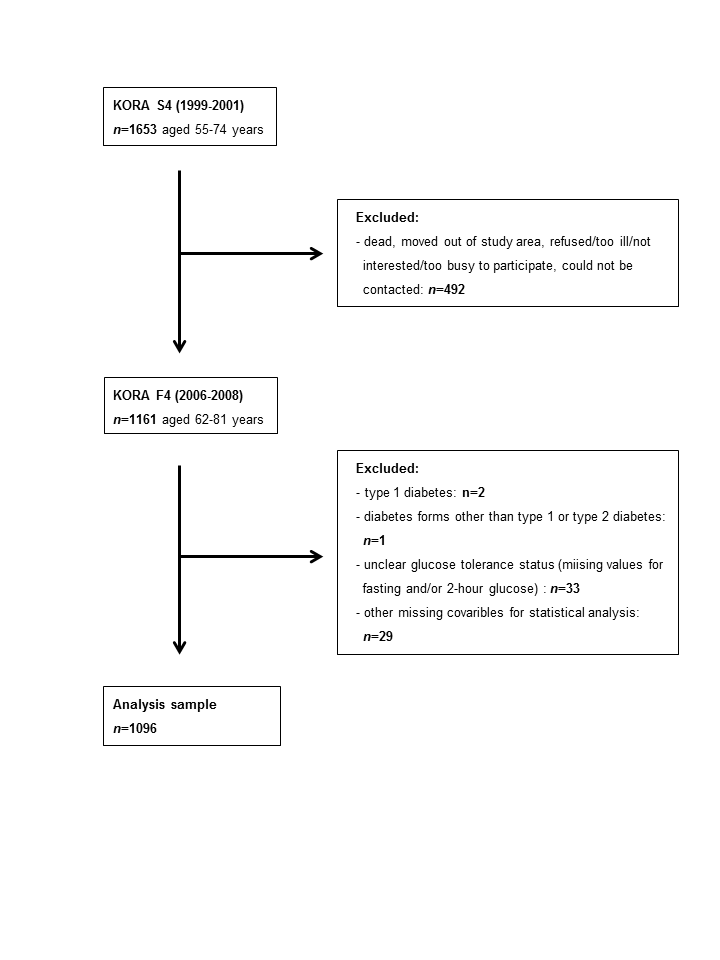

Supplement: Supplementary file 1 — Additional file 1: Table S1. Baseline characteristics (KORA S4, 1999-2001) of participants and non-participants in KORA F4 (2006-2008). Table S2. Association between SFRP5 serum concentrations and glucose tolerance status. Table S3. Association between SFRP5 serum concentrations and HbA1c, duration of diabetes and glucose-lowering medication in study participants with type 2 diabetes. Figure S1. Description of the study design. [file 12933_2017_591_MOESM1_ESM.docx]
